# Massive Hemoperitoneum Secondary to Splenic Laceration After Extracorporeal Shockwave Lithotripsy

**DOI:** 10.7759/cureus.11341

**Published:** 2020-11-05

**Authors:** Ahmed A Salih, Oguz A Turan, Omer Bakal, Andrew Volio, Sabry Ayad

**Affiliations:** 1 Outcome Research, Cleveland Clinic Foundation / Anesthesiology Institute, Cleveland, USA; 2 Outcomes Research, Cleveland Clinic Foundation, Cleveland, USA; 3 Anesthesiology, Cleveland Clinic Foundation, Cleveland, USA; 4 Anesthesiology, Cleveland Clinic Fairview Hospital, Cleveland, USA

**Keywords:** splenic injury, splenectomy, shockwave lithotripsy, splenic rupture, hemoperitoneum, kidney stone

## Abstract

Extracorporeal shock wave lithotripsy (ESWL) is considered a safe technique, but not without complications, though the vast majority are minor complications. We describe a rare case of splenic injury after ESWL.

A 33-year-old male presented to the emergency department (ED) after three weeks experiencing severe intermittent left-sided flank pain that he contributed to a previous motor vehicle accident. Then computerized tomography (CT) revealed a left renal stone. ESWL was performed after three weeks. After being discharged home, he returned the same day to the ED with persistent, worsening abdominal pain, hypotension, and multiple syncopes. CT demonstrated the presence of active contrast extravasation from the spleen likely due to active bleeding. Initial resuscitation was with intravenous fluids and blood products. The following day, the embolization of the splenic artery was done. The patient was discharged home after nine days of conservative management.

After one month, he had shortness of breath due to a large left-sided pleural effusion and lung collapse managed with thoracocentesis and thoracoscopic surgery.

Subsequent follow-up reveals much improvement and successful conservative management.

Splenic injury is a rare complication of ESWL, and all of the 11 reported cases in the literature were managed with splenectomy. Our case is unique in being successfully managed conservatively.

## Introduction

Splenic injuries are frequent after blunt abdominal trauma - the spectrum ranges from a mild laceration to splenic rupture. However, splenic injury secondary to trauma caused by extracorporeal shock wave lithotripsy (ESWL) is rare [1]. The ESWL procedures are used for the treatment of kidney and ureteric stones. It is considered a safe technique, but not without complications, though the vast majority are minor complications. Major complications after ESWL, which cause morbidity or mortality, are uncommon and affect less than 1% of patients undergoing this technique [2].

## Case presentation

**Figure 1:**
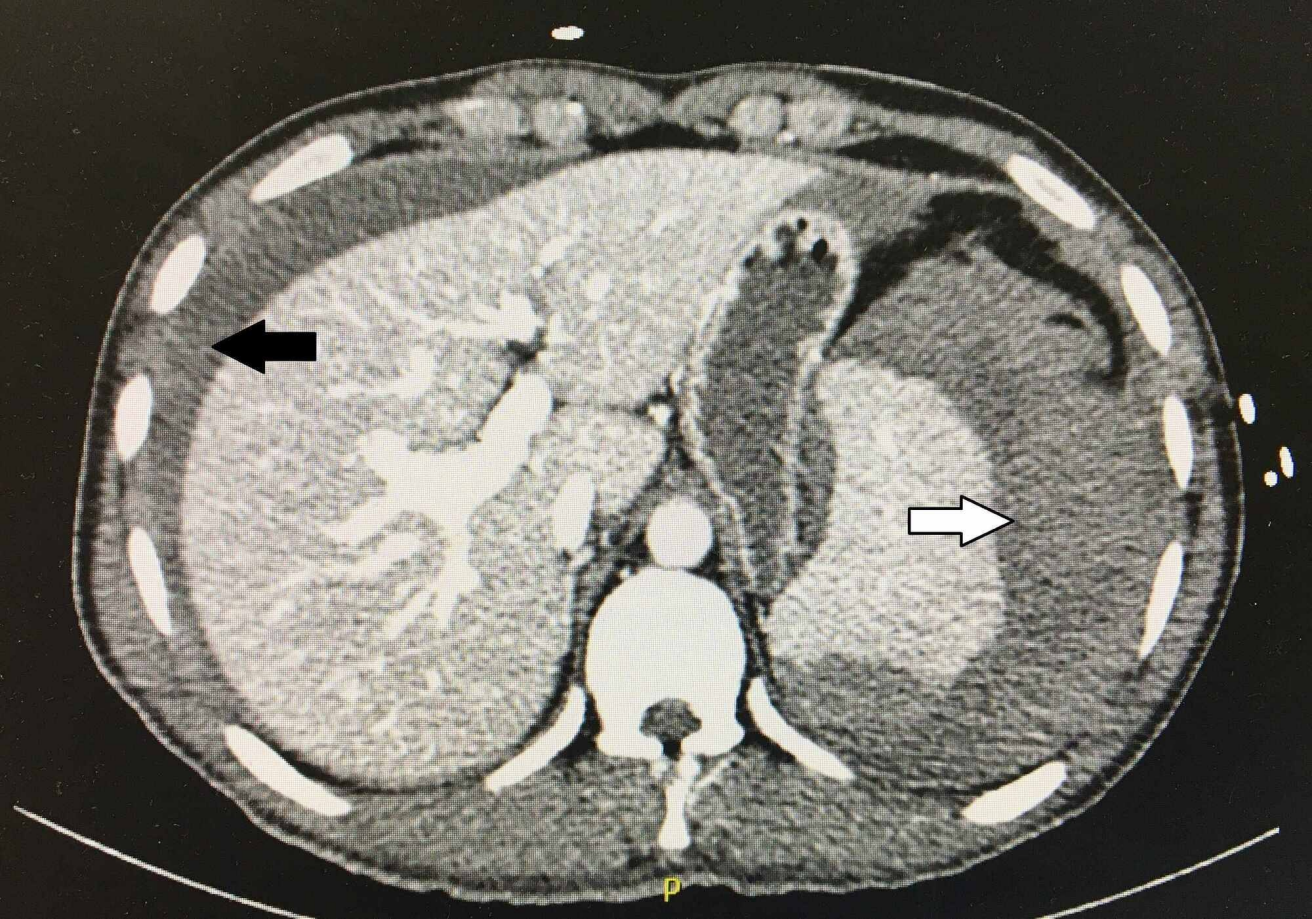
CT of the abdomen showing perisplenic hematoma (white arrow) and hemoperitoneum (black arrow).

**Figure 2:**
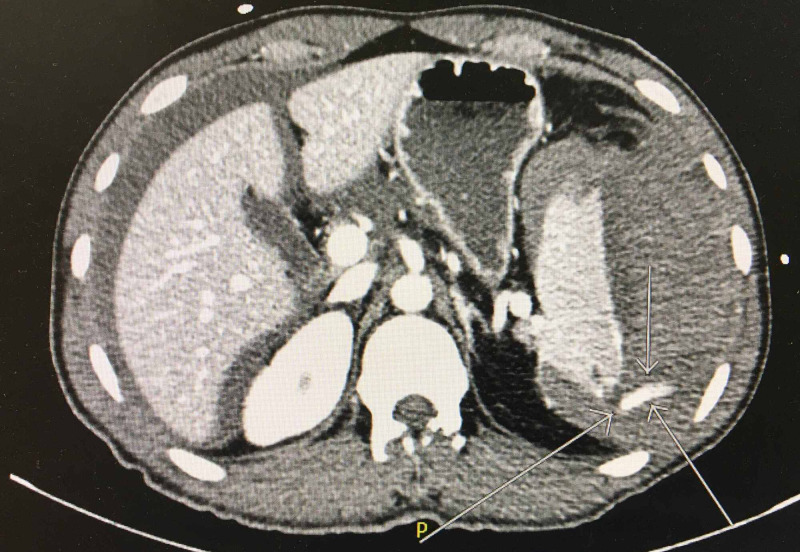
CT showed linear hyper-density extending from the posterior aspect of the spleen (arrows) compatible with active extravasation likely due to active bleeding.

A 33-year-old male with a past medical history of Gilbert's syndrome and chronic mild thrombocytopenia presented to the emergency department (ED) after three weeks of experiencing severe intermittent left-sided flank pain that he was contributed to a motor vehicle accident (MVA) that occurred 47 days before. Computerized tomography (CT) revealed a 9-mm left mid-pole renal stone. ESWL was performed after three weeks under general anesthesia (Propofol 150 mg, Fentanyl 100 µg, Rocuronium 50 mg, endotracheal intubation, and Intermittent Positive Pressure Ventilation). A total of 2500 shocks were delivered 60 shocks per minute and power from 1 to 7 mAmp. A 3-minute pause was conducted after the first 100 shocks. There was a good dispersion of stone on post fluoroscopic imaging. The procedure went uneventful and the patient was then discharged home on the same day in a stable condition. On the same night, he presented to the ED with persistent, worsening abdominal pain, hypotension, and multiple syncopes. CT showed hemoperitoneum, large perisplenic hematoma (Figure 1), and the presence of active contrast extravasation from the spleen likely due to active bleeding (Figure 2). Immediate management included intravenous fluids (Lactated Ringers 75 ml/hr), two units of packed red blood cells (RBC), two units of platelets, two units of fresh frozen plasma (FFP), and Tranexamic acid dose (1000 mg) in the ED and surgical intensive care unit (SICU). The following morning, the patient underwent an interventional radiology (IR) procedure for splenic embolization under monitored anesthesia care (MAC). Then, conservative management continued throughout his hospital stay. After nine days, he was discharged home hemodynamically stable. The patient was instructed strictly to avoid participating in high-risk activities such as contact sports, wrestling, mountain biking, skiing, skydiving, military combat, and vigorous sexual intercourse for three months.

After one month, the patient presented again to the ER complaining of exertional shortness of breath and subacute lower thoracic/epigastric and left upper quadrant abdominal pain. The X-ray and CT showed a large left-sided pleural effusion with small pneumothorax and left lower lung collapse with mass effect, causing a left to right shift of the mediastinum. In the ED, thoracocentesis recovered 2.1 L of exudative fluid. He was treated empirically with vancomycin and Zosyn (Piperacillin / Tazobactam). Pleural fluid was negative for microorganisms and malignant cells. He was admitted to the hospital, and the next day, aspiration of perisplenic hematoma revealed 100 ml of dark old blood. After another two days, imaging showed recurrence of left pleural effusion with collapse/consolidation of the left lower lung. This collapse and effusion was considered "reactive" to the left sub-phrenic hematoma that protruded the diaphragm into the thoracic cavity. On the same day, the IR thoracocentesis was unsuccessful because the fluid was loculated. The patient underwent a left-sided video-assisted thoracoscopic surgery (VATS) with partial pulmonary decortication and effusion drainage. The next day, patient creatinine had risen to 2.72 mg/dl from 1.17 mg/dl the previous day, indicating acute kidney injury (AKI), most likely drug-induced due to vancomycin toxicity, and the co-administration of Zosyn and CT contrast. The serum creatinine was peaked at a value of 5.14 mg/dl and decreased steadily to reach 2.98 mg/dl on his discharge and 1.15 mg/dl on one-month follow-up.

Throughout his stay, the patient continued to have good urine output and acceptable electrolyte levels. The patient was counseled on increasing his oral water intake to 2-3 L a day to maintain hydration and support kidney function. The chest tubes were removed after recording low output, with no subsequent accumulation of pleural fluid. The patient has not reported any dyspnea or shortness of breath. The splenic hematoma continued to be managed conservatively as the latest CT-abdomen showed that this hematoma had shrunk compared to previous imaging one week before. Because of this, the general surgeon decided to hold off on performing a splenectomy. Subsequent follow-up revealed that the patient improved symptomatically, with no further need for surgical intervention.

## Discussion

Recent estimates place the prevalence of nephrolithiasis in the US population at 10.6% for men and 7.1% for women [3]. After its introduction in the 1980s, ESWL has become the standard, convenient, most common, non-invasive procedure treating renal and proximal ureteric calculi. ESWL is a highly effective, and relatively safe minor surgical procedure with a severe life-threatening complication rate of <1% [2].

Progressive pulses are generated by shock waves used in ESWL to induce stone fragmentation by the mean of two mechanisms; direct stress and cavitation. The cavitation mechanism that was used on this patient, is thought to be responsible for complications associated with ESWL [4]. Progressive shock waves enter stones, result in the formation of unstable gas bubbles that violently collapse, causing stone fragmentation [4]. Cavitation will be enhanced as the shock wave rate is increased, so this mechanism may well be involved in local tissue damage at extremely fast shock wave rates [5]. ESWL complications are mainly intrarenal, which can cause parenchymal hemorrhage and subcapsular hematomas and can present as hematuria. Extrarenal complications are less common and include tissue injury, intra-abdominal bleeding/abscess formation, abdominal aorta rupture, pneumothorax, liver, and pancreatic hematomas, pancreatitis, perforation of the bowel, and splenic rupture/hematoma/abscess [5].

To improve the success rates and safety of ESWL, suggestions of new treatment strategies are based on multiple studies of the renal response to shock waves and the mechanisms of shock wave action in stone breakage and renal injury [6]. Better outcomes can be achieved by giving a slower shock wave rate with a stepwise protocol to cause stone comminution with few shock waves and at low power levels as possible, which might also contribute to lower hematoma rates [7].

The choice and success of ESWL are related to several factors that may be evaluated with CT, including stone size, position, and composition, as well as the stone-to-skin-distance (SSD); favorable less than 10 cm [8]. Our patient was thin with a BMI of 21 kg/m^2^ hence the distance from the skin to the kidney was less than 10 cm, making a perfect focusing on the energy bundle possible.

Patients undergoing ESWL with a previous diagnosis of splenic abnormalities, especially splenomegaly, need special care to avoid damage in adjacent organs, as they are more vulnerable to shearing lesions [6]. The CT showed no splenomegaly in this patient.

The splenic laceration is a rare complication of abdomi­nal procedures (mainly after colonoscopy), and also has been reported as a complication of ESWL [9]. Furthermore, both immediate and late-onset splenic rupture are frequent after blunt abdominal trauma [1]. Also, the traumatic splenic rupture in pathological spleens is widely covered. However, the phenomenon of splenic rupture secondary to trauma caused by ESWL is an exceptional occurrence [1]. On a worldwide literature search, there have been only 11 published cases of splenic injury associated with ESWL since the inception of the shock wave technology in 1980 [10-18].

Splenic injury after ESWL can be present as a left upper quadrant or generalized abdominal tenderness, along with left lower chest wall tenderness. Owing to blood loss in the extravascu­lar space, hypotension and even hemorrhagic shock are possible [8].

Imaging plays a central role in the early diagnosis and management of post-ESWL complications. CT of the abdomen may reveal hemoperitoneum, pericapsular splenic hematoma, or splenic rupture. The management depends on the degree of splenic injury and hemodynamic stability [8]. This patient was brought to the ED with a mean arterial blood pressure of 75 mmHg, a pulse rate of 118 bpm, and symptoms compatible with hypovolemic shock. Despite the sizeable intracapsular hematoma, hemodynamic sta­bility was restored shortly. The initial management of this patient was conservative, with strict monitoring in the intensive care unit.

The non-operative management (NOM) is considered the golden standard for treating a patient with blunt splenic trauma (BST), with a success rate of attempt NOM near 90% [19]. All the cases of splenic injury after ESWL described in the literature were treated with operative management (OM), i.e., total splenectomy. None of the patients had conservative management of the splenic trauma, nor was more conservative surgery intervention used [1]. This fact is noticeable, given the high rate of splenic trauma treated conservatively. It is wise to assume that these patients described were reflecting the more severe end of the splenic injury spectrum [19]. Opposite to the 11 cases described in the literature, conservative management was successful in our patient, where the situation of hypovolemia secondary to massive hemoperitoneum due to splenic injury after ESWL responded well to fluid management and blood transfusion in addition to IR and splenic artery embolization.

Research suggests chronic conditions such as hypertension, hyperlipidemia, and coronary artery disease, can contribute to complications associated with ESWL (none of these were present in our patient) [20]. Although no CT scan was done for him shortly after the MVA, there was no intraabdominal hematoma that could be shown on the CT scan three weeks before the ESWL procedure. Also, the laboratory reports of this patient since his MVA showed that the lowest reported platelet count for this patient was 148 k/µL and the highest bilirubin was 3.4 mg/dL with normal liver enzymes and coagulation profiles, hence his past medical history did not affect the medical decision to perform the ESWL. This patient's past medical history of Gilbert's disease and mild thrombocytopenia, besides a significant recent history of MVA and subsequent left upper quadrant/flank abdominal pain for three weeks before the diagnosis of renal stone, raises the suspicion of possible unrecognized minor splenic injury during the MVA that was exacerbated with ESWL intervention (i.e., additional blunt trauma), which makes our case unique in the literature.

The exact mechanism of hematoma formation is not fully understood, but it may be linked to a loss of vascular tensile strength within splenic and renal capsules [20].

## Conclusions

Splenic injury is a rare complication of ESWL and is life-threatening. All the 11 reported cases in the literature were managed with splenectomy. Despite large perisplenic hematoma and associated pleural effusion and AKI, our case is unique in being the first reported splenic injury after ESWL that successfully managed without the need for splenectomy due to the continuing resolution of the hematoma, thanks to the advance diagnostic and interventional radiology and advanced standard of care over the last 40 years.
